# Enter the Royal British College of Nursing

**Published:** 1917-01-27

**Authors:** 


### THE MATRONS' AND SISTERS' DEPARTMENT.

### ENTER THE ROYAL BRITISH COLLEGE OF NURSING.

THE movement for the legal registration of nurses, under the leadership of the Hon. Arthur Stanley, made a very considerable advance last Thursday week, when the members of the Royal British Nurses' Association, in general meeting assembled to the number of 350 or more, resolved *nem. con.* to amalgamate with the College of Nursing, altering at the same time the title of their Association to the "Royal British College of Nursing," of which Her Royal Highness Princess Christian will continue to be President. To give effect to the amalgamation it will be necessary for these bodies to obtain the consent of the Privy Council to a Supplementary Charter and new by-laws, both of which were set out in the summons to the meeting, and are made conditions of the agreement for amalgamation.

A further point is that, so soon as the union is complete, the Royal British College of Nursing shall "promote, or otherwise take steps to obtain, an Act of Parliament for the Registration of Nurses, and the keeping of a Register or Registers of Nurses." This last provision ought finally to set at rest any suspicions, if such are still anywhere genuinely entertained, that the College is really not in earnest about obtaining legal recognition for the nursing profession at the earliest possible opportunity. Hence the supreme importance of every nurse getting her name on to the College Register, and inducing her friends to register, so as to find herself and them, when the time comes, upon the first Register of the Royal British College of Nursing, and so, when the College Registration Bill has become law, upon the first Register of Nurses entitled to vote for their own representatives upon the General Nursing Council.

For every reason, therefore, the Register must go on. The more nurses who enrol, the easier will it be to show the Privy Council and Parliament how overwhelmingly professional opinion is in favour of the movement, and with the more confidence may we face the threatened opposition from a quarter which has been, and still is, consistently hostile to any outside interference with the nurse-training schools, and thus, by pursuing private ends, would render nugatory all efforts through a uniform curriculum and single examination standard for the betterment of the nursing profession at large. Reiterating, then, our urgent advice to nurses to let nothing prevent them from registering, we think we may fairly utilise this momentary lull in the development of events, just while the College and the Royal British Nurses' Association are waiting upon the pleasure of the Privy Council, to give our readers an epitome of the original and Supplementary Charters under which the new incorporation of the "Royal British College of Nursing" will come into existence.

A perusal of the Charter of 1893 shows how many milestones have been passed since those early days. The recital sets out that the Association, founded in 1887, was established "for the purposes of the improvement of the Profession of Nurses and of the promotion of their efficiency and usefulness, and of assisting them by various benevolent schemes." Moreover, "that in furtherance of these objects a List of Nurses has been compiled and published, setting forth the names and addresses of nurses with the names of the hospitals or institutions at which they have been trained, and the length of training which each has received, thus enabling the public to form a more accurate judgment of the professional education and experience of the nurses so registered."

In accordance with this recital the purposes and powers of the Corporation are given as: 1. The founding and maintenance of schemes for the benefit of nurses in the practice of their profession, and in times of adversity, sickness, and old age. 2. The maintenance of an office for supplying information to persons seeking for nurses, and to persons seeking for employment as nurses. 3. The maintenance and publication of a list of persons who may have applied to have their names entered thereon as nurses, and whom the Corporation may think fit to enter thereon, coupled with such information about each person as to the Corporation may seem desirable.

A perusal of the above clauses shows how necessary it had become for the College to make it a condition of amalgamation with the R.B.N.A. that a Supplementary Charter should be granted. This Supplementary Charter extends the purposes of the Corporation so as to include the promotion and the development of nursing as a profession, and of any branch of women's work connected with hospitals, the improvement of the training, education, and professional status of nurses, the promotion of a uniform curriculum and standard of qualification, the institution and conduct of examinations and the granting of diplomas and certificates of proficiency in nursing or any branch of nursing, the making and maintaining of an official Register of persons qualified to act as nurses, and, finally, the promotion of legislation to provide for the State recognition and protection of the Official Register, and for the development and protection of the Profession of Nursing.

Whilst, therefore, the new Corporation takes on all the powers which the College of Nursing had under its Memorandum and Articles of Association, it gets rid of that unbeloved word "Limited"—a stumbling-block and cause of offence to many—and it is able to grant diplomas, and not certificates only, in nursing or any branch of nursing. The members of the new Corporation are the present members of the Royal British Nurses' Association and of the College; all persons who are now on the Register of the R.B.N.A.; and "all persons who from time to time become members in accordance with the by-laws of the Corporation for the time being in force." The new Council consists of the existing Council of the College, and of certain members of the Association, and there is liberty to add six representatives from the Irish Board when formed, thus extending to Ireland equal privileges with Scotland. Branches and local boards may be founded in the United Kingdom or elsewhere. Consultative and examination boards are provided for, and the Council may suspend or expel unworthy members "after full inquiry in such manner as may be prescribed by the by-laws."

So much of our space has been taken up in summarising the provisions of the Charters that, we cannot on this occasion consider the new by-laws of the Corporation except so far as to deal with a statement, of which no doubt more will be heard, made by their Chairman to the Executive Committee of the Society for State Registration of Nurses, and published in last week's number of its Journal.

It is alleged "the professional status of the R.B.N.A would, under the Supplemental Charter, be absorbed into a lay Corporation, as it provided for the inclusion of the laity as members of the College without any qualification."

Under By-law 4 (*b*) "The Council may elect, as members of the Corporation, any persons whom the Council may deem it advisable, in the interests of the Corporation, to elect as members thereof." In terms, therefore, this statement is true, but what is its value? Originally the R.B.N.A. might, and did, admit to membership "any duly qualified medical practitioner, as defined by the Medical Acts." Membership was thus not confined to trained nurses; doctors were admissible and admitted. Presumably the Association had a "professional status," though it admitted doctors and in spite of its President being a "lay" person. The College has lost its "professional status" because laymen may be admitted "in the interests of the Corporation." Is it a fact that doctoring and nursing are one and the same profession? Have nurses no interests to which doctors may be opposed? Are there no doctors who employ, and prefer to employ, nurses whom nobody representative of trained nurses would admit to its registers? What was the attitude of a large number of doctors in the analogous case of the registration of midwives? To ask these questions is to answer them. The plain fact is that "professional registrationists" who are women need the aid of men to help them in finance, in methods of business management, and in many of the multifarious activities which are integral parts of a College of Nursing, and a General Nursing Council. For this reason, no doubt, because they knew the need of such help, the promoters of the Royal British Nurses' Association took power to admit doctors to membership. The Royal British College of Nursing also presumably wants the help of men, but finds no sound reason why it should limit itself to medical men, often, unfortunately, very unbusinesslike folk. Hence the Council has taken power to admit any men or women whom it may deem desirable "in the interests of the Corporation."

To descend from the general to the particular, and to take a concrete example. Who has helped the cause of State Registration more than Mr. Stanley? Yet he, forsooth, as one of the "laity" must be excluded "if the professional status of the R.B.N.A." is not to be "absorbed into a lay Corporation"! Away with such pedantry, or worse!

